# *Neuregulin 1* Expression and Electrophysiological Abnormalities in the *Neuregulin 1* Transmembrane Domain Heterozygous Mutant Mouse

**DOI:** 10.1371/journal.pone.0124114

**Published:** 2015-05-19

**Authors:** Leonora E. Long, Paul Anderson, Elisabeth Frank, Alex Shaw, Shijie Liu, Xu-Feng Huang, Didier Pinault, Tim Karl, Terence J. O’Brien, Cynthia Shannon Weickert, Nigel C. Jones

**Affiliations:** 1 Schizophrenia Research Institute, Sydney, New South Wales, Australia; 2 Neuroscience Research Australia, Randwick, New South Wales, Australia; 3 School of Medical Sciences, University of New South Wales, Sydney, New South Wales, Australia; 4 Department of Medicine (Royal Melbourne Hospital), Melbourne Brain Centre, University of Melbourne, Parkville, Victoria, Australia; 5 University of Wollongong, Wollongong, New South Wales, Australia; 6 INSERM U1114, psychopathologie cognitive et physiopathologie de la schizophrénie, Fédération de Médecine Translationnelle de Strasbourg (FMTS), Université de Strasbourg, Strasbourg, France; 7 School of Psychiatry, University of New South Wales, Sydney, New South Wales, Australia; Kanazawa University, JAPAN

## Abstract

**Background:**

The *Neuregulin 1* transmembrane domain heterozygous mutant (*Nrg1* TM HET) mouse is used to investigate the role of Nrg1 in brain function and schizophrenia-like behavioural phenotypes. However, the molecular alterations in brain Nrg1 expression that underpin the behavioural observations have been assumed, but not directly determined. Here we comprehensively characterise mRNA Nrg1 transcripts throughout development of the *Nrg1* TM HET mouse. In addition, we investigate the regulation of high-frequency (gamma) electrophysiological oscillations in this mutant mouse to associate molecular changes in Nrg1 with a schizophrenia-relevant neurophysiological profile.

**Methods:**

Using exonic probes spanning the cysteine-rich, epidermal growth factor (EGF)-like, transmembrane and intracellular domain encoding regions of *Nrg1*, mRNA levels were measured using qPCR in hippocampus and frontal cortex from male and female *Nrg1 *TM HET and wild type-like (WT) mice throughout development. We also performed electrophysiological recordings in adult mice and analysed gamma oscillatory at baseline, in responses to auditory stimuli and to ketamine.

**Results:**

In both hippocampus and cortex, *Nrg1* TM HET mice show significantly reduced expression of the exon encoding the transmembrane domain of *Nrg1 *compared with WT, but unaltered mRNA expression encoding the extracellular bioactive EGF-like and the cysteine-rich (type III) domains, and development-specific and region-specific reductions in the mRNA encoding the intracellular domain. Hippocampal Nrg1 protein expression was not altered, but NMDA receptor NR2B subunit phosphorylation was lower in *Nrg1 *TM HET mice. We identified elevated ongoing and reduced sensory-evoked gamma power in *Nrg1* TM HET mice.

**Interpretation:**

We found no evidence to support the claim that the *Nrg1* TM HET mouse represents a simple haploinsufficient model. Further research is required to explore the possibility that mutation results in a gain of Nrg1 function.

## Introduction

Genome-wide association studies have identified *neuregulin 1* (*NRG1*) as a candidate risk gene for schizophrenia [1–5]. Schizophrenia is characterised by increased expression of *NRG1* splice variants and altered isoform expression ratios, which are linked to polymorphisms in intronic and promoter regions of the gene [6–9], possibly via high nucleotide diversity in the regulatory regions of *NRG1* leading to increased molecular change [9]. The leading Nrg1 mouse studied in the context of a ‘schizophrenia’ model is the heterozygous transmembrane domain (*Nrg1* TM HET) mouse [1,10], which is implied to be a hemizygous ‘knockout’ model of reduced Nrg1 function. However, examination of the type and location of the transmembrane domain (TM) mutation suggests this may not be the case, and a decrease in brain Nrg1 synthesis has not been demonstrated. An understanding of how Nrg1 expression and neurobiological function are changed in *Nrg1* TM HET mice is critical to determine which aspects of the Nrg1-related molecular and cellular changes found in schizophrenia may be recapitulated in this mouse, and to better model the relationships between Nrg1 alterations and pathophysiological/behavioural outcomes.

*NRG1* contains a bioactive epidermal growth factor (EGF)-like domain that binds to ErbB tyrosine kinase receptors [11–15]. Protease cleavage of the membrane precursor or secretion as a soluble form allows extracellular diffusion of NRG1 [16–23]. In the *Nrg1* TM HET mouse, a NEO cassette replaces the TMD of the *Nrg1* gene, resulting in heterozygous deletion of the TM DNA [1]. *Nrg1* TM HET mice show behavioural features relevant to the symptoms of schizophrenia [1,10,24–27] and display altered susceptibility to the neurobehavioural effects of cannabinoids and other psychoactive drugs [28–35]. However, the neurobiological changes underlying this schizophrenia-like phenotype are not known.

Abnormal high frequency cortical oscillations (gamma; 30–80 Hz) are emerging as a common neurophysiological phenotype present in schizophrenia [36]. These oscillations have been associated with various cognitive functions, including attention, memory and perception [37–39], which are disrupted in patients with schizophrenia [40]. One of the most consistent pathologies in the cortex of patients with schizophrenia involves GABAergic interneurons, which are dependent on NRG1-ErbB4 signaling for maturation [41]. GABAergic interneurons expressing the calcium-binding protein parvalbumin (PV) contribute to the generation of gamma oscillations [42], and postmortem studies of schizophrenia patients have identified robust molecular deficits in the PV+ subset of cortical interneurons [43–45]. These findings lead to the hypothesis that abnormal gamma oscillations occurring in schizophrenia result from aberrant perisomatic inhibition of pyramidal neurons mediated by PV-positive interneurons. Several lines of clinical and experimental evidence suggest that this synaptic dysfunction involves a hyporegulation of NMDA receptors [46–48], although the precise mechanisms involved remain unclear [49].

Nrg1 regulates GABAergic interneuron and NMDA receptor function, and may therefore also have a role in gamma oscillations. The receptor for Nrg1, ErbB4, is highly expressed on PV-positive interneurons [41,50], and Nrg1-ErbB4 signalling alters intrinsic excitability and evoked GABA release of these cells [41,50–52]. Nrg1 signalling can also affect glutamatergic synapse development and plasticity [53]. Moreover, ErbB4 receptors attach to the scaffolding protein PSD-95 in the same location as NMDA receptor NR2 subunits [54,55], and Nrg1-ErbB4 signalling stimulates NMDA receptor NR2B subunit phosphorylation, which may be necessary for synaptic plasticity (e.g. long-term potentiation) [56]. Given this involvement of Nrg1-ErbB4 signalling in the development and function of NMDA receptors and inhibitory interneurons, abnormal Nrg1 expression, such as that which may exist in the *Nrg1* TM HET mouse model, might in turn lead to abnormal gamma oscillations. Indeed, ErbB4 mutation leads to altered gamma oscillations [57,58].

Here, we describe the expression of *Nrg1* mRNA across postnatal life in two schizophrenia-relevant brain regions that express high levels of Nrg1. We hypothesised that transcription of the 5’ region containing the ‘bioactive’ EGF-like domain would be intact in *Nrg1* TM HET mice, given that the exon coding for the EGF-like domain is located upstream from the TM DNA deletion. Furthermore, we hypothesised that *Nrg1* TM HET mice would show reduced expression of TM and intracellular *Nrg1* mRNA. We also describe our measurement of the impact of Nrg1 TMD mutation on cortical gamma oscillatory activity, at baseline and in response to the non-competitive NMDA receptor antagonist ketamine, and on the expression and phosphorylation of NMDA receptor subunit mRNA and protein. We hypothesized that *Nrg1* TM HET mice would exhibit abnormal gamma oscillations.

## Materials and Methods

### Ethics statement

All research and animal care procedures were approved by the University of New South Wales Animal Care and Ethics Committee (#10/98B), or by the University of Melbourne Animal Ethics Committee (#1011868), in accordance with the Australian Code of Practice for the Care and Use of Animals for Scientific Purposes.

### Animals

Heterozygous *Nrg1* mutation was achieved in *Nrg1* TM HET mice by replacing the transmembrane domain DNA with DNA for three premature stop codons in each reading frame, a transcription termination signal, and a neomycin resistance gene under control of a phosphoglycerate kinase promoter [1]. *Nrg1* TM HET and wild type-like control *Nrg1* ^+/+^ (WT) mice were housed with their dams in litters prior to weaning on postnatal day (PND) 21, after which mice were pair-housed with limited environmental enrichment (mouse igloo (Bioserv, Frenchtown, USA) and a metal ring in the cage lid [10]) under a 12:12 h light:dark schedule. Food and water were available *ad libitum*. Genotypes were confirmed after weaning using tail tip biopsy and PCR amplification (primers: forward 5’-GCTAGCTTGTTATTTATGCTTAAAG-3’; WT reverse 5’-CCACCACACACATGATGCCGAC-3’; *Nrg1* TM HET reverse 5’-GCACAGTCGAGGCTGATCAGCG-3’). For gene expression studies, tissue was collected from male and female *Nrg1* TM HET and WT mice from 5–11 different litters per age group (postnatal day (PND) 7, 10, 14, 21, 28, 35, 49 and 161; Table 1). For electrophysiology studies, adult female *Nrg1* TM HET and WT mice (~12–16 weeks old) were transported to the Department of Medicine (Royal Melbourne Hospital), University of Melbourne (n = 20 *Nrg1* TM HET, n = 22 WT). Some of these mice were then used for locomotor studies (n = 8 *Nrg1* TM HET, n = 14 WT), while others were used for protein measurement (n = 12 *Nrg1* TM HET, n = 13 WT).

**Table 1:** Developmental cohort used for qPCR experiments. RIN expressed as mean ± S.E.M.

Postnatal Day	Genotype	Gender	RIN	n
*Hippocampus*				
7	12 WT, 10 HET	11 F, 11 M	9.63 ± 0.47	22
10	12 WT, 12 HET	12 F, 12 M	9.70 ± 0.35	24
14	12 WT, 11 HET	12 F, 11 M	9.24 ± 0.33	23
21	12 WT, 12 HET	12 F, 12 M	9.10 ± 0.25	24
28	12 WT, 12 HET	12 F, 12 M	8.88 ± 0.27	24
35	12 WT, 11 HET	12 F, 11 M	8.73 ± 0.36	23
49	10 WT, 11 HET	11 F, 10 M	8.73 ± 0.46	21
161	10 WT, 10 HET	9 F, 11 M	8.77 ± 0.46	20
*Prelimbic cortex*				
7	9 WT, 7 HET	9 F, 7 M	9.61 ± 0.54	16
10	9 WT, 7 HET	8 F, 8 M	9.48 ± 0.57	15
14	9 WT, 8 HET	10 F, 7 M	9.30 ± 0.53	17
21	9 WT, 7 HET	9 F, 7 M	8.61 ± 1.09	16
28	12 WT, 11 HET	11 F, 12 M	8.75 ± 0.58	23
35	12 WT, 10 HET	11 F, 11 M	8.60 ± 0.76	22
49	9 WT, 8 HET	7 F, 10 M	8.55 ± 0.51	17
161	11 WT, 9 HET	9 F, 11 M	8.81 ± 0.31	20

### RNA extraction

Total RNA was extracted for qPCR analysis from the left hippocampus (4.3–18.3 mg; n = 181) and prelimbic cortex (1.3–11.7 mg; n = 147) of male and female *Nrg1* TM HET and WT mice at PND 7, 10, 14, 21, 28, 35 and 161 using TRIzol, following the manufacturer’s protocol (Invitrogen, Carlsbad, CA, USA). Total RNA was resuspended in DEPC-treated water after purification by precipitation (Sigma-Aldrich, Castle Hill, NSW, Australia). The yield of total RNA was analysed using a spectrophotometer (Nanodrop ND-1000; Thermo Scientific, Wilmington, DE, USA). The quality of total RNA was determined using an Agilent Bioanalyzer 2100 (Agilent Technologies, Palo Alto, CA, USA): 100–200 ng RNA was applied to an RNA 6000 Nano LabChip, without heating prior to loading. The RNA integrity number (RIN) was used as an indicator of RNA quality, ranging from 1–10 (lowest—highest quality). Samples had an average RIN of 9.03 ± 0.10 (Table 1). Any sample with a RIN ≤ 6.5 was excluded from qPCR experiments.

### Reverse transcription and quantitative PCR

**Fig 1:**
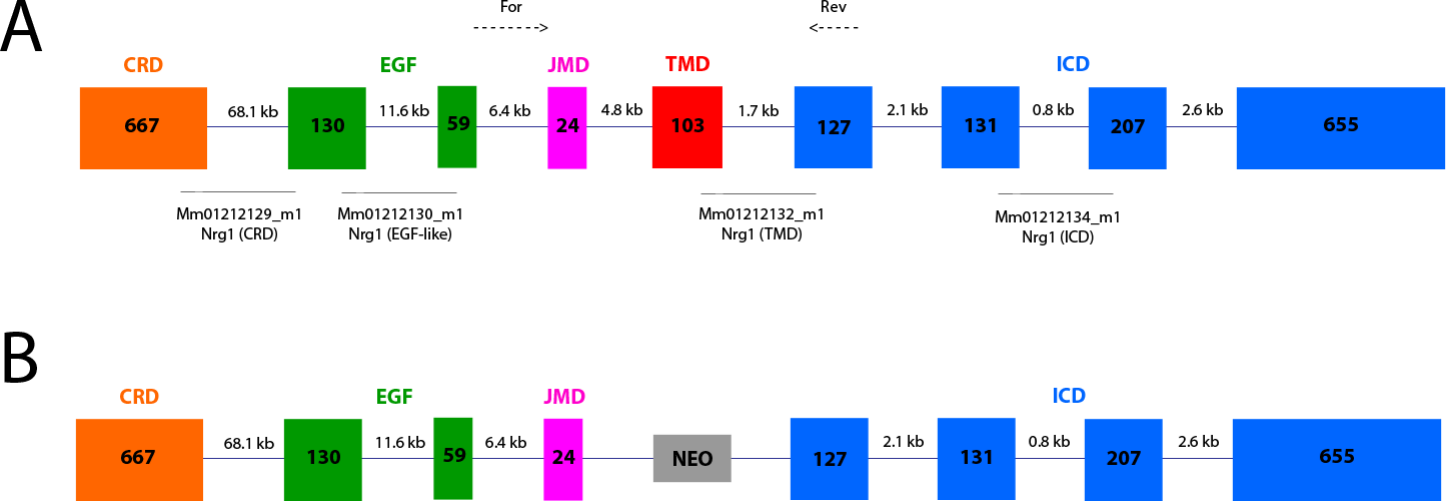
Schematic representation of mouse *Nrg1* type III mRNA. Structures are shown as **A)** wild type structure and **B)** with the transmembrane domain replaced by the NEO cassette. Solid lines indicate the exons spanned by the assay probes in the Applied Biosystems Taqman gene expression assays. Dotted lines indicate the positions of the forward and reverse primers used in endpoint PCR amplification and sequencing of *Nrg1* transcripts. CRD = cysteine-rich domain; EGF = EGF-like (‘bioactive’) domain; JMD = juxtamembrane domain; TMD = transmembrane domain; ICD = intracellular domains

cDNA was synthesised in two reactions of 0.5–2 μg of total RNA using the Superscript III First-Strand Synthesis Kit (Invitrogen) with random hexamers according to the manufacturer’s protocol. Pre-designed TaqMan Gene Expression Assays (Applied Biosystems, Foster City, CA, USA) were chosen for four distinct segments of the *Nrg1* transcript, the pan-*ErbB4* transcript, five genes encoding the NMDAR subunits of interest, and three housekeeper control transcripts (Fig 1A; Table 2). qPCR was performed with an ABI Prism 7900HT Fast real-time PCR system with a 384-well format. The PCR reaction was initiated by uracil-DNA glycosylase treatment for 2 min at 50°C and denaturation for 10 min at 95°C, followed by 40 cycles consisting of heating to 95°C for 15 s followed by annealing and extension at 60°C for 1 min. Measurements were performed in duplicate and relative quantities determined from a seven-point standard curve. Control wells containing no cDNA template displayed no amplification. Efficiencies of the qPCR reactions ranged from 63% to 100%, and r^2^ values were between 0.96 and 1.00. Outliers were excluded from qPCR analysis if their normalised expression values were greater than two standard deviations from the group mean. Expression levels were normalised to the geometric mean of three reference genes (Table 2).

**Table 2:** Applied Biosystems TaqMan gene expression assay numbers.

Gene Name	Gene Symbol	Taqman Assay
*Genes of Interest*		
Nrg1 (RefSeq: NM_178591.2) exons 1–2 (cysteine rich domain)	Nrg1 (CRD)	Mm01212129_m1
Nrg1 (RefSeq: NM_178591.2) exons 2–3 (extracellular EGF-like domain)	Nrg1 (EGF-like)	Mm01212130_m1
Nrg1 (RefSeq: NM_178591.2) exons 5–6 (transmembrane domain)	Nrg1 (TMD)	Mm01212132_m1
Nrg1 (RefSeq: NM_178591.2) exons 7–8 (intracellular domain)	Nrg1 (ICD)	Mm01212134_m1
*Reference Genes*		
TATA box binding protein	Tbp	Mm00446973_m1
Ubiquitin C	Ubc	Mm01201237_m1
Eukaryotic 18S rRNA	18S	Hs99999901_s1

### Sequencing

For qualitative examination of the *Nrg1* transcript, cDNA samples from the left hippocampus of female HET and WT mice aged PND 49 (n = 2/genotype) were amplified using 40 cycles of endpoint PCR with 5U Red Hot DNA polymerase (Thermo Scientific). Primers were designed to amplify potential splice variants of type III *Nrg1* spanning exon 3 to exon 6 (Fig 1A and 1B; NCBI reference sequence NM_178591.2; forward 5’-AGCTTCTACAAGCATCTTGGGAT-3’, annealing temperature 64°C; reverse 5’-GTGAGGGCCATTCGCTATGT-3’, annealing temperature 66.1°C). PCR products were separated on a 3% agarose gel at 80 V for 75 min. Bands of interest corresponding to amplicons that either omitted or incorporated the TMD (predicted sizes 118 and 225 bp respectively) were excised and DNA was extracted and purified using a QIAquick Gel Extraction Kit (Qiagen) and eluted into 10 mM Tris buffer, ph 8.5. DNA concentration was measured using a Nanodrop ND-1000 spectrophotometer (Thermo Scientific). PCR product sequencing was performed in forward and reverse, with the same primers used in the endpoint PCR, using 3–5 ng DNA added to a DNA sequencing kit (Big Dye Terminator v3.1 Cycle Sequencing, Applied Biosystems). Cloning and sequencing was performed by direct ligation of PCR products into the pGEM-T Easy vector, followed by sequencing using the T7 primer. All sequencing was performed on an Applied Biosystems 3730 DNA Analyser at the Ramaciotti Centre for Gene Function Analysis (University of New South Wales, Sydney, Australia).

### Bioinformatic analysis

Alignment of cDNA sequences was performed using LALIGN (http://embnet.vital-it.ch/software/LALIGN_form.html [59]). RNA sequencing data [60] was obtained from the European Nucleotide Archive (accession: GSE39866) and aligned to the mouse genome (mm10) using Tophat version 1.5.0 with default parameters. The July 2007 mouse genome reference assembly (NCBI37/mm9) was scanned for alternative transcription start sites within the *Nrg1* gene by looking for conserved elements within multiple mammalian genomes and for transcription factor binding sites within ENCODE data sets using the UCSC Genome Browser. *In silico* translation of predicted mRNA sequences in *Nrg1* TM HET mice and WT controls was carried out with CLC Genomics Workbench 5.5.1 (CLC Bio, Aarhus, Denmark).

### Nrg1 Western blotting

The right hippocampus of male and female *Nrg1* TM HET and WT littermates aged PND 35 (n = 11 per genotype and sex) was homogenised by grinding with a pestle in buffer (0.1 M Tris pH 7.5, 50% glycerol, 0.0053 mM aprotinin, 1:100 protease inhibitor cocktail P8340; Sigma, St Louis, MO, USA) and diluted to 2 mg/ml after protein concentration determination by Bradford assay (Sigma). Proteins were heated (5 min, 95°C), loaded alongside a molecular weight ladder (Precision Plus, Bio-Rad, Hercules, CA, USA) onto 8–12% gradient SDS polyacrylamide gels and electrophoresed for 80 min at 120 V. Standard curves were generated by loading 1–40 ug of crude protein homogenate, and sample analysis was conducted using 5 ug of protein homogenate. Electrophoresed proteins were transferred onto nitrocellulose membranes (Bio-Rad) at 100 V for 2 h, which were then blocked for 2 h at 4°C in Tris-buffered saline (TBS) containing 0.1% Tween 20 and 5% skim milk (Bio-Rad) and incubated overnight at 4°C in primary rabbit anti-Nrg1 (1:200, sc-348, Santa Cruz Biotechnology, Dallas, TX, USA) or rabbit anti-β-actin antibody (1:5000, #4967, Cell Signalling, Danvers, MA, USA). The following day, blots were incubated in goat anti-rabbit IgG peroxidase-conjugated secondary antibody (1:1000–1:2000, AP132P, Millipore, Billerica, MA, USA) for 1 h at room temperature. Immunoreactive bands were detected using enhanced chemiluminescence reagent (Millipore) and exposed to film (Amersham Hyperfilm, GE Healthcare Australia, Rydalmere, NSW, Australia). The blots were scanned and the optical density measured by Image J (National Institutes of Health, http://rsb.info.nih.gov/ij/). Immunoreactivity of Nrg1 protein was normalised to the β-actin band detected in the same lane, and an internal control (pooled sample from entire cohort) loaded onto the same gel. The specificity of analysed immunoreaction signals using the anti-Nrg1 antibody sc-348 was previously confirmed in pre-absorption experiments in which antibodies were pre-incubated with a five-fold excess of their respective epitope-containing peptides or of a non-related peptide [6]. Furthermore, this antibody (sc-348) against Nrg1 has been shown to detect this protein in brain, as it detects a significant (30–75%) reduction, depending on the brain region, of Nrg1 protein levels in floxed Nrg1 mutant mice designed to knock down Nrg1 postnatally. Also in support of the ability of this antibody (sc-348) to recognise Nrg1 protein is the observation that in a transgenic Nrg1 overexpression mouse, levels of Nrg1 in brain detected with this antibody were increased four-fold [61].

### Electrode implantation surgery

Mice were implanted with cranial recording electrodes for the acquisition of surface electrocorticograms (ECoG). Briefly, animals were anaesthetised using isoflurane (Abbott Pharmaceuticals, USA) and placed in a stereotactic frame. A single midline incision was made, four holes were drilled in the skull, and epidural brass recording electrodes were implanted 2 mm anterior and 2 mm lateral to bregma bilaterally (active electrodes) and 1.5 mm posterior and 1 mm lateral to lambda bilaterally (reference and control electrodes, respectively).

### Electrophysiology recordings and analyses

ECoG recordings of ongoing parietal cortical activity were undertaken in a quiet, dimly lit behavioural testing suite in a first cohort of mice, as previously described [62]. Mice (n = 12 *Nrg1* TM HET; 8 WT) were habituated to the environment for 30 min, during which they were in a state of quiet wakefulness. A 30 min baseline ECoG recording was then acquired, which was followed by injection with either ketamine (10 mg/kg subcutaneous (s.c.); Parnell Laboratories, Australia) or saline (10 ml/kg s.c.) and a subsequent 60 min of recording. Animals remained awake for the entire recording. A second cohort of mice underwent recording to assess auditory-evoked activity (n = 8 *Nrg1* TM HET; 14 WT); recording was done in SR-Lab startle chambers and stimuli generated with SR-Lab software (San Diego Instruments, CA, USA). Stimuli consisted of 85 dB white noise pulses of 10 ms duration and an inter-stimulus interval of 6 s; background white noise was maintained at 70 dB. These stimuli were below the threshold to elicit a startle response in mice of both genotypes. In both cohorts, ECoG recordings were acquired using a Powerlab 4/30 amplifier and A-D converter and LabChart 7 software (AD Instruments, Australia), sampled at 2000 Hz, and band pass filtered offline at 0.5–500 Hz. Data were exported to MATLAB software and visually inspected for movement artifacts; spectral power and response to auditory stimuli were then analysed. Ongoing gamma activity was measured using fast Fourier transformations (Hamming window, 0.48 Hz resolution) for each two-minute interval of the recording. Total relative power in each frequency band was calculated as the ratio of the raw power of the band divided by the total power (1–100 Hz), with the average relative power defined as the relative power per Hz; e.g., for the theta band (4–8 Hz) P_theta_ = P(4–8 Hz)/P(1–100 Hz). Epochs spanning ± 500 ms from the auditory stimulus were extracted, and event related spectral activity was calculated using the EEGLAB toolbox [63]. Power was calculated using Morlet wavelets ranging from 3 to 10 cycles across 20–200 Hz.

### Locomotor activity

Locomotor effects of ketamine were also assessed in this second cohort of mice at least 2 weeks after electrophysiology experiments. Mice were individually placed in photocell tracking chambers (Med Associates, St Albans, VT, USA) to measure spontaneous baseline activity, and after 30 min were administered ketamine (10mg/kg, s.c.) or saline and allowed to explore the chamber for a further 60 min. Quantification of the distance travelled both before and after injection was objectively assessed using Activity Monitor software (Med Associates, St Albans, VT, USA).

### NR2B Western blotting

Between 1–2 weeks after electrophysiology experiments, female *Nrg1* TM HET and WT mice from the first electrophysiology cohort were randomised to receive an injection of either ketamine (10 mg/kg s.c) or saline (10 ml/kg s.c). 10 min later, mice were sacrificed via cervical dislocation, the brains rapidly removed, and the prelimbic cortex manually dissected, snap-frozen in liquid nitrogen, and stored at -80°C. For protein extraction, tissue samples were ground on dry ice, the powder was dissolved in RIPA buffer (150 mM NaCl, 50mM TRIS, 0.1% SDS, 1% sodium deoxycholate, 1% Triton X-100, Roche Complete Protease Inhibitor Cocktail and Sigma Phosphatase Inhibitor Cocktail) then spun at 12,000g for 20 min at 4°C, and the supernatants were collected. Protein concentration of the supernatants was determined with a BCA protein assay kit and adjusted to a concentration of 1 mg/ml total protein in SDS loading buffer. After heating at 95°C for 5 min, proteins were separated on a 6% SDS polyacrylamide gel. The protein bands were electrophoretically transferred to PVDF membranes and immunoblotted with anti-NMDA NR2B (Invitrogen, mouse monoclonal, 1:2000) or anti-NMDA NR2B pY1472 (Novus Biological, rabbit polyclonal, 1:2000) antibodies. Immunoreactive bands were detected with a chemiluminescent substrate kit and exposed to X-ray film. The blots on the X-ray film were scanned and the sum optical density quantitatively analysed with Image J. The immunoreactivities of proteins were normalized against α-tubulin (Sigma, mouse monoclonal, 1:10000). All data were expressed as relative levels of the saline-treated WT mean.

### Statistical analysis

Statistical analyses were conducted with SPSS Statistics 20 (IBM, NY, USA) or Graphpad Prism 5 (La Jolla, CA, USA). For the electrophysiology studies, spectral power and specific frequency bands were compared using Mann Whitney U-tests (for between genotype analyses). The magnitude of the ketamine-induced increase in gamma power was quantified as the area under the curve and compared using Student’s t-test. Locomotor activity was assessed in 2 min time bins, and compared between genotypes both before and after ketamine injection using two-way analysis of variance (ANOVA), as per our previous work [64]. For gamma frequency event-related spectral perturbations (ERSP), individual trials were averaged into 2 min blocks (20 trials in each), and comparisons between drug conditions were made using the mean of 100 trials spanning 5–15 min post injection, and statistical significance was assessed with one-way repeated measures ANOVA. The relationship between ongoing gamma and ERSP was assessed with Pearson’s correlation.

For molecular studies, differences in normalised mRNA expression were tested with three-way ANOVA, with genotype, sex and age (PND) as the grouping variables, followed by post-hoc t-tests to identify differences between genotypes at specific ages and by Fisher’s least significant difference (LSD) post-hoc analyses to identify specific differences between age groups. Differences in normalised Nrg1 protein expression were tested with two-way ANOVA with genotype and sex as the grouping variables. Differences in normalised NMDAR NR2B subunit protein expression were tested with two-way ANOVA, with genotype and ketamine treatment as the grouping variables, followed by post-hoc t-tests to identify differences between these variables. For all analyses, a significant result was indicated when p ≤ 0.05.

## Results

### *Nrg1* transmembrane domain mRNA is reduced in *Nrg1* TM HET mice but the mRNA encoding the bioactive EGF-like domain is not reduced

We examined the influence of *Nrg1* TMD mutation on mRNA expression of *Nrg1* exons across postnatal development. The geometric mean of the expression of Ubc and Tbp mRNA and 18S rRNA did not change significantly over this age range or with regard to genotype in the hippocampus or prelimbic cortex (S1 Fig). *Nrg1* mRNA expression did not differ between male and female mice in either brain region (three-way ANOVA main effect for sex: p > 0.05).

**Fig 2:**
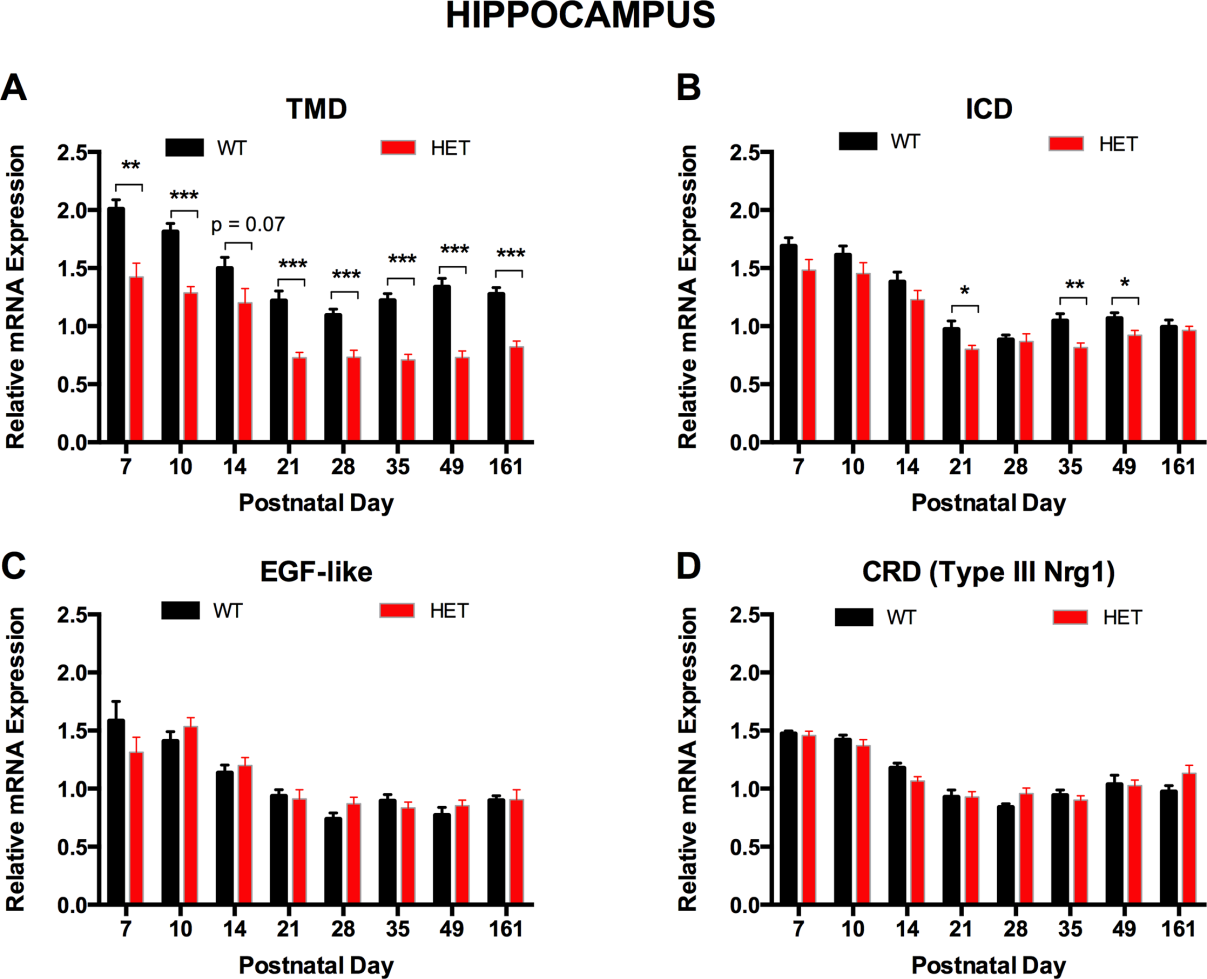
*Nrg1* mRNA expression in hippocampus of *Nrg1* TM HET mice and WT controls. Expression of mRNA transcribed from exons encoding the **A)** transmembrane (TMD), **B)** intracellular (ICD), **C)** extracellular EGF-like and **D)** cysteine-rich (CRD) domains of *Nrg1* was determined by qPCR (y-axis, mean (+ S.E.M.) expression normalised to the geometric mean of three reference genes) and plotted by postnatal day. * p < 0.05, ** p < 0.01, *** p < 0.001 (ANOVA)

**Fig 3:**
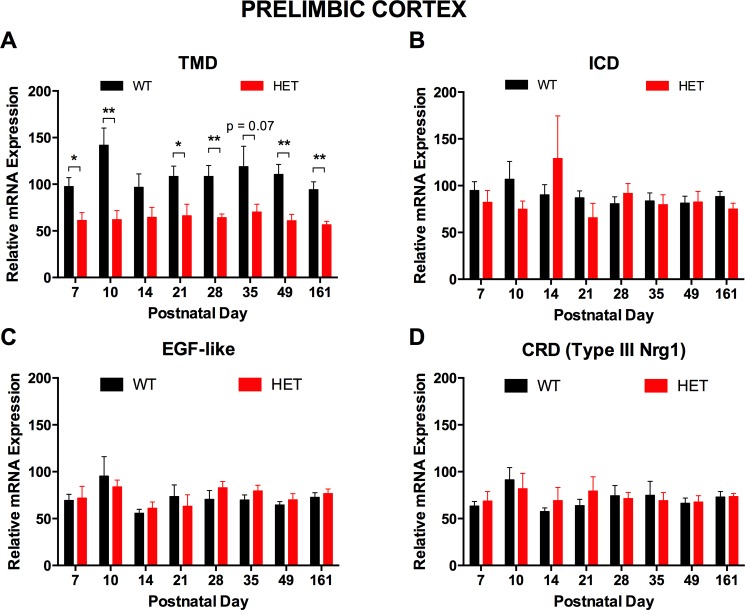
*Nrg1* mRNA expression in prelimbic cortex of *Nrg1* TM HET mice and WT controls. Expression of mRNA transcribed from exons encoding the **A)** transmembrane (TMD) and **B)** intracellular (ICD), **C)** extracellular EGF-like and **D)** cysteine-rich (CRD) domains of *Nrg1* was determined by qPCR (y-axis, mean (+ S.E.M.) expression normalised to the geometric mean of three reference genes) and plotted by postnatal day. * p < 0.05, ** p < 0.01 (ANOVA)

We observed robust reduction in expression of *Nrg1* TMD mRNA in *Nrg1* TM HET mice. Expression was decreased across postnatal development by at least 20% and up to 45% in the hippocampus [three-way ANOVA main effect for genotype: F(1, 134) = 169.23, p < 0.001; Fig 2A] and by at least 34% and up to 56% in the prelimbic cortex of *Nrg1* TM HET mice [three-way ANOVA for genotype: F(1, 109) = 46.52, p < 0.001; Fig 3A]. Expression of mRNA for the intracellular domain (ICD) was reduced by 13–22% at postnatal days 21, 35 and 49 in the hippocampus of *Nrg1* TM HET mice [three-way ANOVA for genotype: F(1, 141) = 18.56, p < 0.001; Fig 2B], but was not significantly altered in the prelimbic cortex at any age [F(1, 110) = 0.24, p > 0.05; Fig 3B]. Levels of mRNA coding for the extracellular EGF-like domain and type III *Nrg1*-specific cysteine-rich domain (CRD) were not significantly altered by the *Nrg1* TMD mutation in either region [three-way ANOVA for genotype in hippocampus: EGF-like domain F(1, 123) = 0.21, p > 0.05; CRD F(1, 142) = 0.11, p > 0.05; Fig 2C and 2D; in prelimbic cortex: EGF-like domain F(1, 109) = 0.09, p > 0.05; CRD F(1, 107) = 0.33, p > 0.05; Fig 3C and 3D].

In the hippocampus, all *Nrg1* exons measured showed higher expression in early life before decreasing to a plateau by PND 21 [three-way ANOVA main effect for age: TMD F(7, 134) = 29.41, p < 0.001; ICD F(7, 141) = 41.85, p < 0.001; EGF-like domain F(7, 123) = 25.06, p < 0.001; CRD F(7, 142) = 37.34, p < 0.001]; *Nrg1* CRD mRNA expression underwent an additional increase at PND 49. Expression of all *Nrg1* exons measured in the prelimbic cortex remained stable across postnatal development [three-way ANOVA main effect for age: TMD F(7, 109) = 0.78, p > 0.05; ICD F(7, 110) = 0.89, p > 0.05; EGF-like domain F(7, 109) = 1.51, p > 0.05; CRD F(7, 107) = 0.694, p > 0.05].

### No evidence for a splice variant skipping the exon encoding the transmembrane domain in wild type-like or *Nrg1* TM HET mice

**Fig 4:**
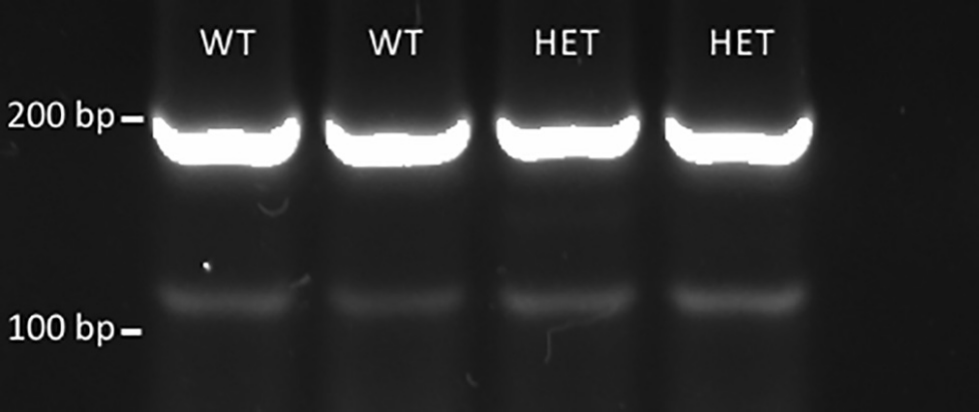
Representative image of PCR products amplified from cDNA of hippocampus of *Nrg1* TM HET mice and WT controls at postnatal day 49.

Since the regions of the *Nrg1* transcript on either side of the TMD appeared intact, we tested if a *Nrg1* splice variant that lacks the TMD could be detected in mouse brain. We investigated whether there are novel splice variants in WT or *Nrg1* TM HET mice that exclude the TMD (exon 5 in NCBI reference sequence NM_178591.2; Fig 1A) of the most brain-abundant type III *Nrg1* [65]). We performed PCR with primers spanning exon 3 and exon 6 of NM_178591.2, predicting two products of 118 (exon 5 exclusive) and 225 bp (exon 5 inclusive). Sequencing of the two products yielded by our PCR (Fig 4; S1 Table) showed that the larger products (195 – 283 bp) amplified from the forward primer aligned with bases 876–1073 of the reference sequence NM_178591.2, including exon 5, in both WT and *Nrg1* TM HET mice. The smaller, fainter product could not be directly sequenced; however, following cloning and sequencing it was found to align to an intergenic region unrelated to the *Nrg1* locus (data not shown).

To further explore the potential expression of novel *Nrg1* splice variants that omit the TMD, we also performed bioinformatic analysis of previously sequenced samples of embryonic mouse cerebral cortex [60]. These datasets included exon junctions from a range of known *Nrg1* transcripts, but there was no evidence of a transcript including an exon junction between exons 3 or 4 and exon 6. This suggests that the TMD may not be typically spliced out when the mRNA encoding the intracellular domain is included in the transcript.

### Nrg1 protein expression is not altered in *Nrg1* TM HET mice

**Fig 5:**
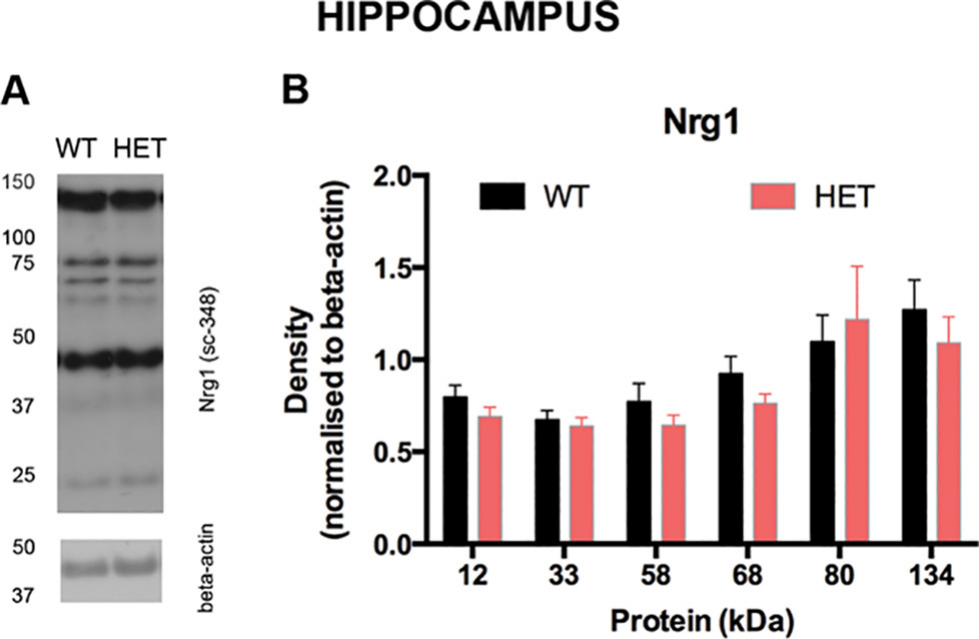
Nrg1 protein expression in hippocampus of *Nrg1* TM HET mice and WT controls at postnatal day 35. **A)** Representative Western blot image. A molecular weight ladder is shown on the left of this figure. **B)** Mean (+ S.E.M.) optical density (normalised to beta-actin) of Nrg1 protein fragments.

Western blot with an antibody to the C-terminal of Nrg1 in the hippocampus of *Nrg1* TM HET mice and WT controls aged postnatal day 35 showed immunoreactive bands at 11, 33, 58, 68, 80 and 134 kDa. The 134 and 58 kDa bands are roughly consistent with the sizes of bands previously inferred to be proprotein and cytoplasmic fragments, respectively, of β1a isoforms of type III Nrg1 [66], while the 80 kDa band is consistent with the calculated size of 77 kDa of the protein translated from the reference sequence for mouse type III Nrg1 NM_178591.2. Standard curves are shown in S2 Fig. There was no effect of genotype or sex on the density of any of these bands (two-way ANOVA for genotype and sex: p > 0.05; Fig 5).

### *Nrg1* TM HET mice have elevated ongoing gamma frequency power

**Fig 6:**
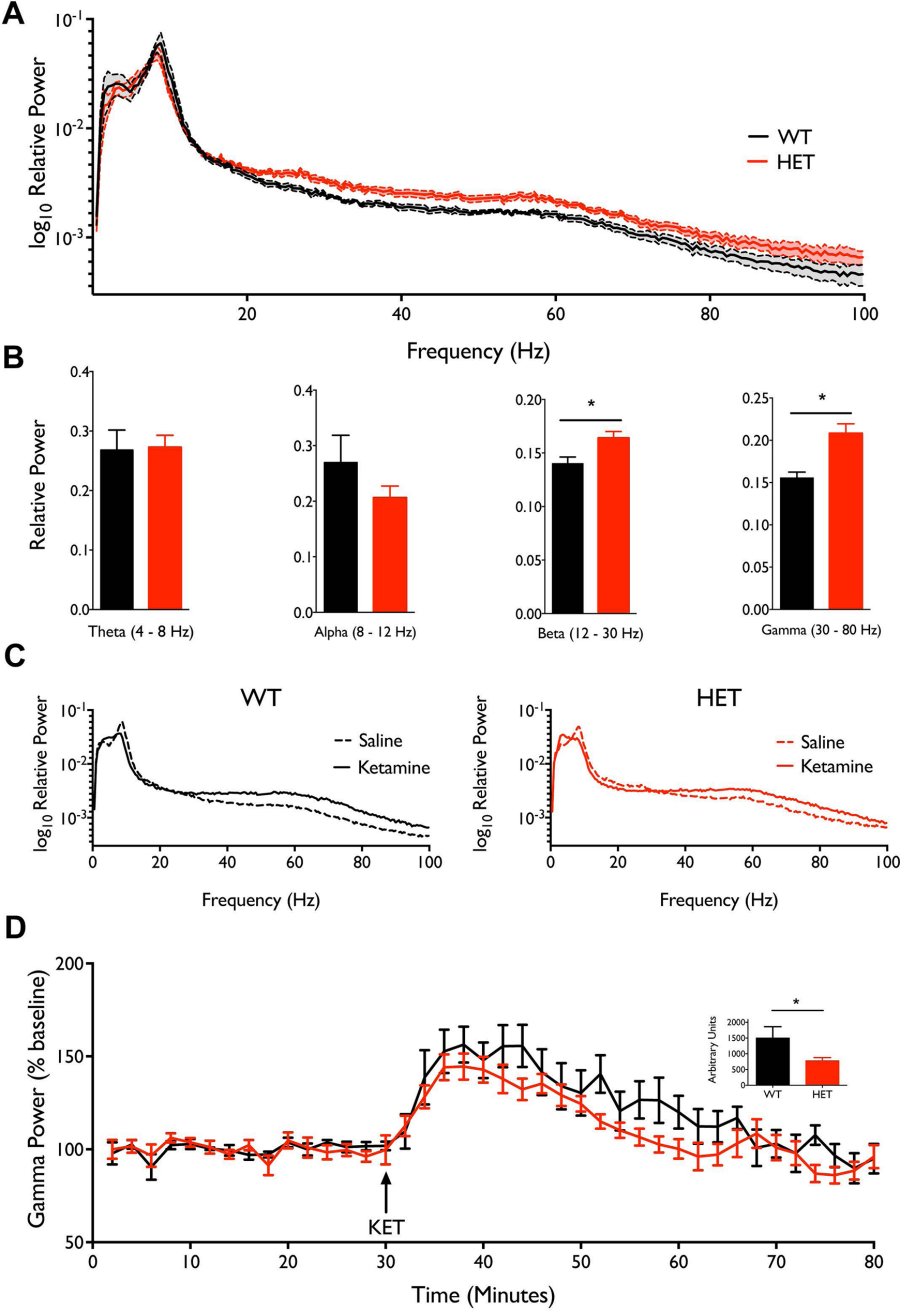
ECoG recordings in awake, freely moving mice show that spontaneous high frequency cortical oscillations are increased in *Nrg1* TM HET mice. **A)** Relative power 0.5–100 Hz in *Nrg1* TM HET (n = 7) and WT (n = 5) mice shows increased power at higher frequencies. Analysis performed on recordings during the 0–30 minutes post-saline injection; data displays the average power of 15 individual two-minute epochs. Spectra significantly differ (p = 0.0012). **B)** Relative ECoG power in theta, alpha, beta and gamma bands. Power was significantly higher in *Nrg1* TM HET mice in the beta and gamma bands (*p < 0.05). **C)** Relative spectral power following saline or ketamine injection in *Nrg1* TM HET mice and WT mice. **D)** Gamma power following ketamine administration, normalised to percentage of mean power in the 30 min preceding injection. **Inset)** Area under the curve from 30–60 min, quantifying changes in gamma power; *p < 0.05. All data are presented as mean ± SEM.

Spectral analysis of ongoing ECoG recordings revealed a persistent elevation in spectral power in *Nrg1* TM HET mice compared to WT (Fig 6A). This abnormal ECoG activity was selectively observed in the higher frequency bands, beginning at 20 Hz (β frequency) and appearing most predominantly in the gamma frequency range (30–80 Hz; Fig 6B). This was showed to be statistically significant when comparing the relative power of the gamma (*Nrg1* TM HET = 0.21 ± 0.01; WT = 0.16 ± 0.01; Mann-Whitney U = 2, p = 0.01) and beta (*Nrg1* TM HET = 0.16 ± 0.01; WT = 0.14 ± 0.01; U = 4; p = < 0.05) frequency bands in *Nrg1* TM HET and WT mice. No significant differences between genotypes were observed in the theta (4–8 Hz) or alpha (8–12 Hz) frequency bands.

### *Nrg1* TM HET mice display reduced gamma frequency response to ketamine

We next investigated the cortical oscillatory dynamics of *Nrg1* TM HET mice in response to ketamine. All mice exhibited a typical electrophysiological response to ketamine with a pronounced increase in the power of ongoing gamma oscillations (Fig 6C and 6D). In *Nrg1* TM HET mice, the magnitude of this effect was smaller (maximum change from baseline: 144% in *Nrg1* TM HET vs. 156% in WT) and shorter acting (effect returning to baseline at 26 min post-ketamine injection in *Nrg1* TM HET vs. 36 min in WT). This response to ketamine was quantified by calculating the area under the curve (Fig 6D inset), which was significantly smaller in *Nrg1* TM HET mice (t = 2.14, p < 0.05). Saline injection did not affect ongoing gamma power in either genotype (data not shown).

### *Nrg1* TM HET mice show reduced auditory-evoked gamma oscillations

**Fig 7:**
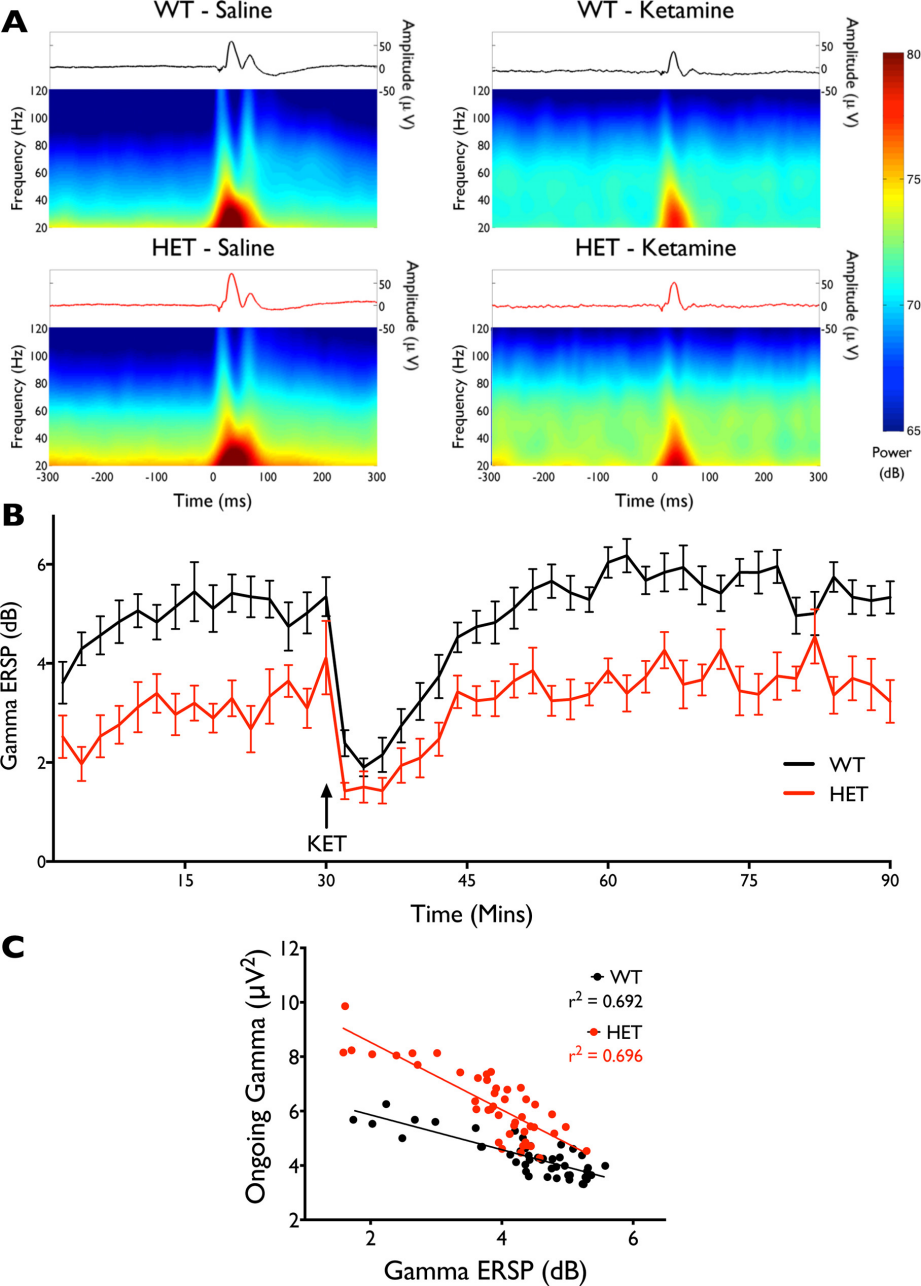
Reduced event-related gamma activity in *Nrg1* TM HET mice and ketamine disruption of sensory responses. **A)** Grand average evoked potentials from WT and *Nrg1* TM HET mice under both saline and ketamine conditions (top panels) and heatmaps representing the event-related spectral perturbation (ERSP) triggered by auditory stimuli (bottom panels). Averages were generated from all trials over 2–12 min post-injection. Note the increased gamma power prior to stimulus in *Nrg1* TM HET mice relative to WT, and in the ketamine conditions. **B)** Gamma frequency (30–80 Hz) ERSP following auditory stimulus (0–60 ms post-stimuli) over time; data points represent 2 min mean ± SEM. **C)** Correlations between ongoing gamma activity and gamma ERSP; data points are 2 min means.

We next examined the ECoG response elicited by auditory stimulation in *Nrg1* TM HET and WT mice (Fig 7). The electrographic response (Fig 7A) in control WT mice appeared to have three components: an early, large positive deflection, a late positive short-lasting wave, and a subsequent long-lasting negative wave. Ketamine treatment abolished the late positive short-lasting wave of the evoked potential and also the subsequent long-lasting negative wave. When quantifying the gamma power evoked by the stimulus, *Nrg1* TM HET mice exhibited reduced sensory-evoked gamma power compared to WT controls [F (3, 142) = 116.8, p < 0.0001; Fig 7B]. Ketamine also reduced the gamma response to the auditory stimuli [F (1, 32) = 100.71, p < 0.01], although there was no interaction between drug and genotype. Correlation analysis showed significant negative correlations between the power of ongoing gamma oscillations and the event-related gamma signal for both genotypes (r = -0.83 for both WT and Nrg1 TM HET mice; Fig 7C), such that the higher the ongoing gamma oscillations, the smaller the evoked gamma responses.

### *Nrg1* TM HET mice exhibit hyperlocomotion, but genotype does not alter the locomotor impact of ketamine

**Fig 8:**
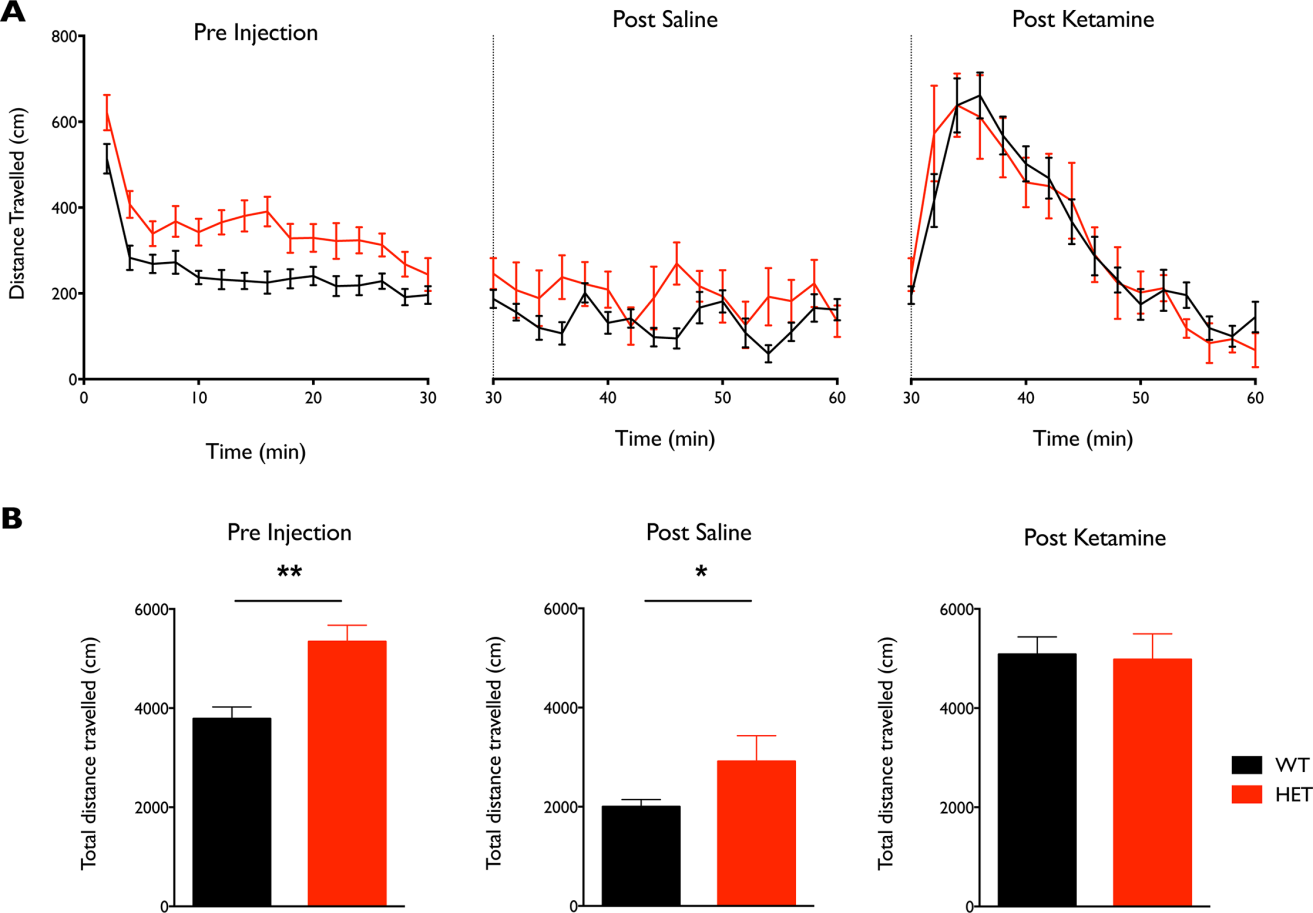
A) Locomotor activity of *Nrg1* TM HET mice and WT controls over 30 min periods: upon introduction to recording chamber, following saline, and following ketamine injection. **B)** Total distance travelled in the 30 min periods prior to and following ketamine and saline administration; * p < 0.05, **p < 0.001.

Consistent with previous reports, *Nrg1* TM HET mice displayed hyperlocomotion upon being introduced to the testing chamber, travelling a significantly greater distance compared with WT controls (t = 3.89; p < 0.001; Fig 8). This hyperlocomotion was still present in *Nrg1* TM HET mice following saline administration, but was less pronounced (t = 2.13; p < 0.05). Ketamine administration elicited higher locomotor activity compared with saline in both WT and *Nrg1* TM HET mice (F (1, 20) = 37.22; p < 0.0001), but there was no significant difference in locomotion in *Nrg1* TM HET compared with WT mice after ketamine administration (t = 0.17; p = 0.87).

### Cortical NMDA receptor subunit mRNA expression is not altered in *Nrg1* TM HET mice

**Fig 9:**
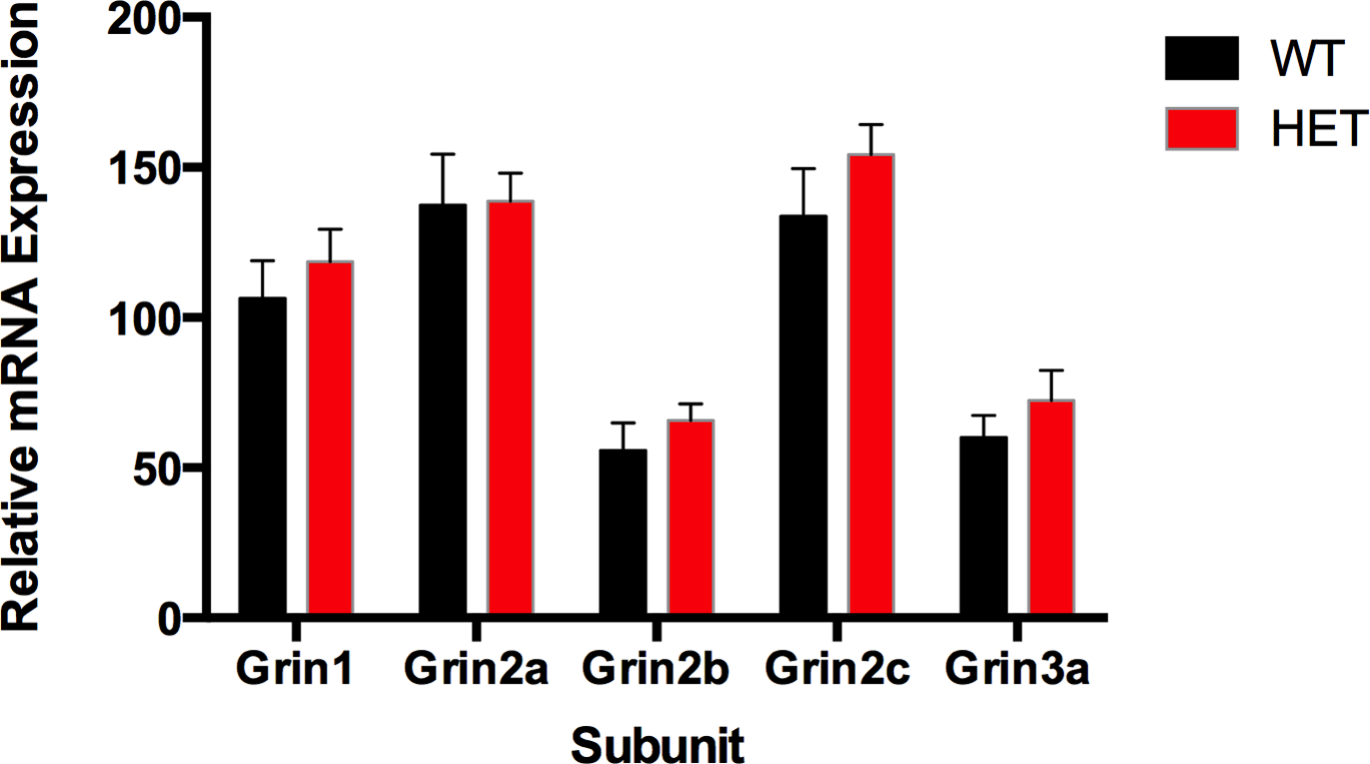
NMDAR subunit mRNA expression in prelimbic cortex of *Nrg1* TM HET mice and WT controls. Expression of mRNA transcribed from exons encoding the NMDAR subunits was determined by qPCR (y-axis, mean (+ S.E.M.) expression normalised to the geometric mean of three reference genes).

Since Nrg1 signalling can alter gene expression, we examined whether the mechanisms behind altered cortical gamma power in *Nrg1* TM HET mice involve changes in cortical mRNA expression of the NMDA receptor subunits NR1, NR2A, NR2B, NR2C and NR3A. We observed no significant differences in mRNA expression of any of these subunits between adult (PND 161) *Nrg1* TM HET and WT mice (two-way ANOVA main effect for genotype: p > 0.05; Fig 9) or between male and female mice (main effect for sex: p > 0.05).

### Cortical NR2B receptor phosphorylation is reduced in *Nrg1* TM HET mice

**Fig 10:**
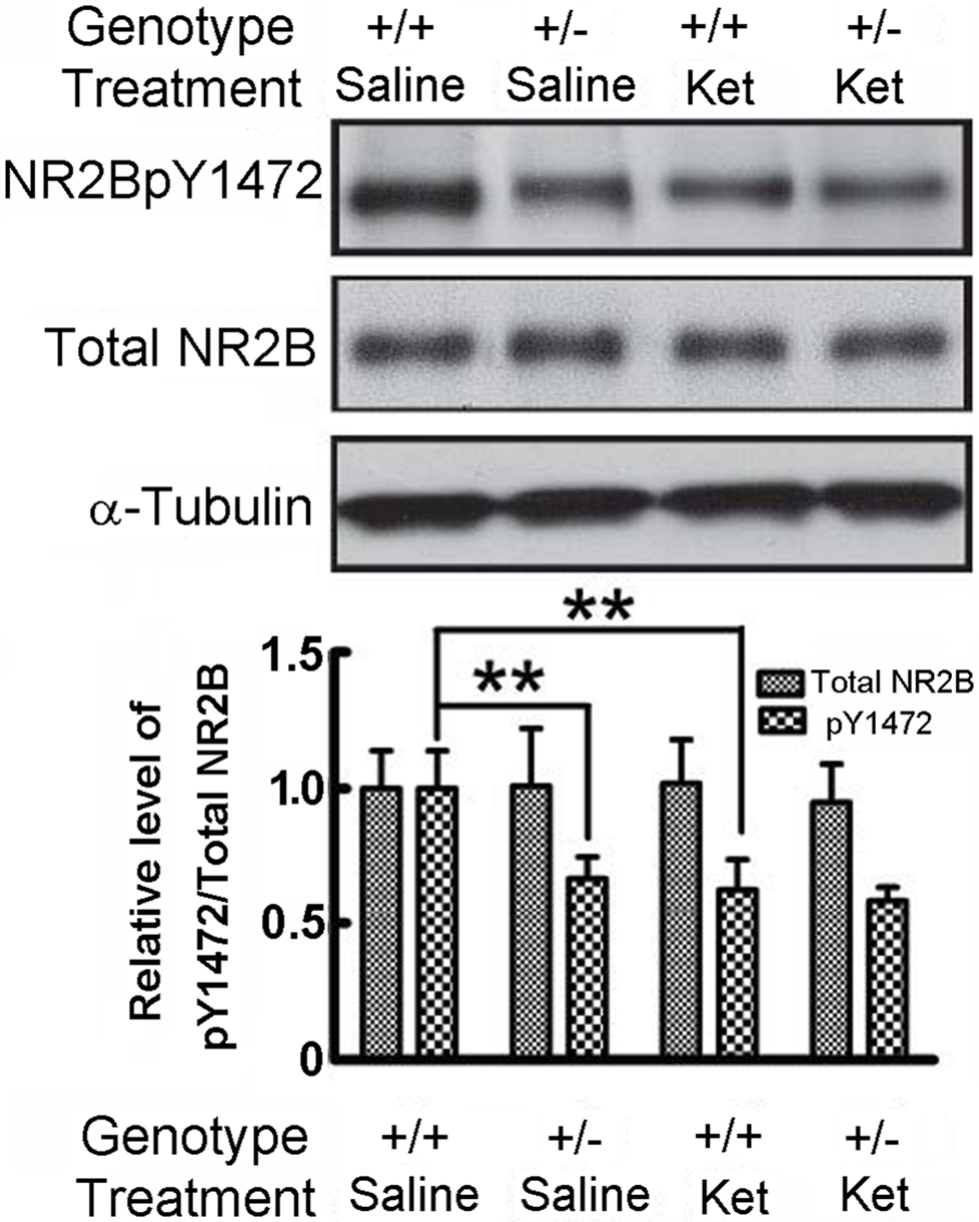
Phosphorylation of NMDA NR2B subunits is reduced in *Nrg1* TM HET mice, but the ketamine-induced reduction in NR2B phosphorylation observed in WT mice is not apparent in the mutants. **A)** Representative Western blot image. **B)** Total cortical NR2B protein is not affected by genotype or treatment, but phosphorylation of the NR2B Y1472 residue is reduced in *Nrg1* TM HET mice. Ketamine reduces phosphorylation in WT, but not in *Nrg1* TM HET mice (**p<0.01).

We next tested whether the activation state of the NMDAR was changed, by examining the phosphorylation of the Y1472 residue of the NR2B subunit in the cortex (Fig 10). We found reduced phosphorylation of Y1472 in *Nrg1* TM HET mice compared to WT mice [two-way ANOVA for genotype: F (1, 20) = 20.25, p < 0.001]. We also identified a significant main effect of ketamine [two-way ANOVA for treatment: F (1, 20) = 37.52, p < 0.0001] such that ketamine reduced phosphorylation of NR2B in both genotypes, and a significant genotype x treatment interaction [F (1, 20) = 13.61, p < 0.01] such that saline-treated WT mice showed significantly higher levels of NR2B phosphorylation than either saline-treated *Nrg1* TM HET mice or WT or *Nrg1* TM HET mice treated with ketamine. We found no differences between any groups in the total expression of NR2B receptor protein [two-way ANOVA for genotype: F (1, 20) = 0.79, p > 0.05; treatment: F (1, 20) = 0.09, p > 0.05], consistent with the mRNA expression data.

## Discussion

Here we show for the first time that expression of the mRNA encoding the bioactive EGF-like domain of *Nrg1* is unaltered in *Nrg1* TM HET mice. As expected, we found reduced expression of mRNA for the transmembrane domain of *Nrg1*; however, interestingly, we found that expression of the intracellular domain of *Nrg1*, downstream of the TMD mutation, is not dramatically or consistently altered at the mRNA or protein level. Table 3 summarises the expected and observed changes in each *Nrg1* exon examined. While the *Nrg1* TM HET mouse has previously been conceptualised as a globally hypomorphic model of Nrg1 function, our data suggest that in fact, it may be inaccurate to conceptualise this mouse as a pure loss of Nrg1 function or as a simple *Nrg1* ‘knock-out’ model. A second major finding of this study is that *Nrg1* TM HET mice show increased gamma power in ongoing cortical oscillations, but reduced sensory-evoked gamma responses, in addition to reduced phosphorylation of the NMDA receptor NR2B subunit. These findings are consistent with the idea of a gain of Nrg1 function and an associated abnormal increase in gamma power at baseline, and may provide molecular and neurophysiological correlates of the behavioural abnormalities previously observed in this model.

**Table 3:** Observed and expected changes in expression of Nrg1 exons.

Transcript	Expected	Observed
Nrg1 (CRD)	No change	No change
Nrg1 (EGF-like)	No change	No change
Nrg1 (TMD)	50% reduction	20–45% reduction in hippocampus; 34–56% reduction in prelimbic cortex
Nrg1 (ICD)	50% reduction	Transient, 13–22% reduction in hippocampus; no change in prelimbic cortex

### Nrg1 expression in *Nrg1* TM HET mice

The expression of all *Nrg1* exons measured in the hippocampus in *Nrg1* TM HET mice decreases across postnatal life, but appears to remain relatively stable in the prelimbic cortex. In humans, *Nrg1* mRNA is developmentally regulated in the prefrontal cortex, as *Nrg1* is expressed at higher levels in pre- and early postnatal life before decreasing to adult levels [67,68], consistent with our hippocampal results. However, our data suggest that there may be region- and species-specific elements involved in the developmental regulation of *Nrg1* gene expression in the prefrontal cortex.

As expected, mRNA expression of the TMD of *Nrg1* was decreased in *Nrg1* TM HET mice in both hippocampus and prelimbic cortex, which is consistent with the location of the mutation in the gene. Importantly, there was no change in expression of the EGF-like, bioactive extracellular domain mRNA that is present in all *Nrg1* isoforms. This finding, combined with the reduced TMD mRNA expression, challenges previous assumptions that the *Nrg1* TM HET mouse results in a loss of Nrg1 function [1] due to loss of Nrg1 mRNA synthesis. Furthermore, this finding does not rule out the idea that more soluble Nrg1 could be available for signalling in the *Nrg1 TM HET* mouse. This idea of increased Nrg1 function in this mouse may not be surprising given that the EGF-like domain is upstream of the TMD DNA excision and the known *Nrg1* promoter regions are left intact by the genetic manipulation and Nrg1 splice variants lacking mRNA encoding the TM exist normally. Moreover, we did not observe any change in mRNA expression of the cysteine-rich domain that characterises the type III *Nrg1* isoform family, and which is also upstream of the DNA mutation. Another unexpected result is that mRNA encoding the intracellular domain of *Nrg1*, downstream of the gene mutation, is unaffected in the prelimbic cortex and only reduced at transient time points in the hippocampus. Furthermore, our measurement of Nrg1 protein using an antibody directed at the C-terminus did not reveal any alterations in protein expression in mutant mice. An intriguing possibility suggested by these observations is that more of the Nrg1 intracellular domain exists in a form that is untethered to the cell membrane and may be ‘free’ to back-signal to the nucleus [69], thereby representing a possible gain of intracellular Nrg1 function.

There are at least three possible molecular explanations for the expression of wild type-like levels of intracellular regions of Nrg1 mRNA, which are downstream from the TMD mutation: 1) alternative splicing, 2) internal promoter usage, or 3) allelic compensation. We investigated the possibility of alternative splicing such that the TMD exon is skipped in some *Nrg1* transcripts. We sequenced a shorter fragment amplified from exon 3 and exon 6 of the mouse type III *Nrg1* sequence, but this did not contain the expected Nrg1 sequence, suggesting that the putative ΔTM is not expressed in our samples. Bioinformatic analysis of RNA sequencing data from samples of wild type mouse embryonic cortex [60] further suggested that a ΔTM *Nrg1* splice variant is not normally produced. However, data from wild type samples does not rule out the possibility that RNA sequencing in the *Nrg1* TM HET mouse would reveal an enriched amount of a variant that was not detectable by our endpoint PCR methods. The design of the mutation, in which a transcription stop site was introduced, suggests that alternative splicing is an unlikely explanation for the maintenance of wild type-like ICD mRNA expression in *Nrg1* TM HET mice, although it is possible that the neomycin resistance cassette facilitates cryptic splicing, whereby a novel product that includes the NEO cassette as well as the ICD could be produced. A second explanation for the relatively unchanged levels of Nrg1 intracellular domain mRNA is that there are novel transcription start sites that allow transcription to begin downstream from the TMD mutation. However, while several intronic regions of the *Nrg1* genomic sequence are conserved between mammalian species, there is little evidence for putative novel promoters, based on the near absence of potential transcription factor binding sites either just upstream of, or downstream of the TMD mutation (determined by DNAse I hypersensitivity or digital genomic footprinting data obtained from ENCODE). A third possibility is that there is allelic compensation, such that synthesis of the wild type allele is up-regulated in *Nrg1* TM HET mice. This possibility is not supported by the clear and significant decrease in expression of the mRNA encoding the TMD itself, which would not occur if the wild type allele were compensating for the allele containing the deletion. In sum, none of the three possible mechanisms to explain unaltered extracellular and unaltered intracellular Nrg1 mRNA levels in mice heterozygous for the TMD deletion is supported by our investigations, suggesting that *Nrg1* mRNA regulation in brain is considerably complex and requires further research to understand more fully.

Western blot of hippocampal protein with a C-terminal Nrg1 antibody showed several immunoreactive bands. We did not observe any difference between *Nrg1* TM HET and WT mice in the pattern or extent of Nrg1 immunoreactivity corresponding to full-length or putatively cleaved Nrg1 protein. This was unexpected, since replacement of the transmembrane domain with a NEO cassette included the insertion of three stop codons and we expected to see lower expression of full-length Nrg1. Even if there were a novel splice variant lacking the transmembrane domain exon, removal of this exon of 103 bp would be expected to result in a reading frame shift, resulting in impaired translation downstream of the mutation. Indeed, *in silico* translation of the predicted mRNA sequence of such a splice variant revealed a translation stop codon 66 bp downstream from the juxtamembrane region. While there are a number of possible open reading frames downstream of this stop signal, correct splicing and translation into functional protein emanating from the DNA strand containing the genetic TM deletion seems unlikely. Overall, our data suggest a possible compensatory post-transcriptional up-regulation of Nrg1 protein or lack of appropriate degradation and turnover of Nrg1 protein in *Nrg1* TM HET mutant mice such that steady state levels of Nrg1 may be maintained. Future research using antibodies to other parts of the Nrg1 protein, e.g. the N-terminal, will provide an extended the description of the protein changes in *Nrg1* TM HET mutant mice.

### Changes in gamma oscillations in *Nrg1* TM HET mice

We measured gamma frequency oscillations in *Nrg1* TM HET mice in order to observe the impact of the Nrg1 mutation on an electrophysiological measure of neuronal function that has been related to schizophrenia and to inhibitory interneuron circuits, the development of which is governed by Nrg1 signalling. We observed elevated gamma frequency power in *Nrg1* TM HET mice in baseline recordings and concurrent reductions in sensory-driven gamma responses. This supports suggestions from animal studies [70,71] and studies in people with schizophrenia [72] that elevations in baseline gamma activity represent increased ‘noise’ in neural networks, which may impair sensory-evoked gamma oscillations and information processing.

The mechanism(s) underlying the abnormal gamma frequency activity in *Nrg1* TM HET mice is not clear. Nrg1 is a trophic factor that plays a role in several developmental processes, such as interneuron migration [73] and synapse development and maintenance [41,51,53]. Moreover, considerable evidence suggests that Nrg1 signalling affects NMDA receptor function. ErbB4 and NMDA receptors attach to the synaptic scaffolding protein PSD-95 in the same location [55], facilitating direct physical interaction between the two systems. Therefore, alterations in Nrg1 signalling are well placed to influence the development of neuronal circuitry and the regulation of gamma oscillations in the adult mouse. Existing literature supports this: exogenous Nrg1 increases the power of hippocampal gamma oscillation power in hippocampal slices [58,74]. This phenotype is comparable to what is observed in the current study, suggesting that Nrg1 signalling is, at the very least, not reduced in the *Nrg1* TM HET mouse. In contrast, mice with a heterozygous deletion of the Nrg1 ‘bioactive’ EGF-like domain have reductions in the power of stimulus-induced 70 Hz oscillations (but not in other frequencies in the gamma range) [75]. A phenotype of reduced stimulus-evoked gamma oscillations is also observed in the *Nrg1* TM HET mouse in our study, but the protocols between the two studies vary considerably, and no changes in ongoing power were reported in the Nrg1 heterozygous mouse [75]. It should also be noted that the Nrg1 EGF-like mutant mouse is clearly a loss of function model in terms of its mutation being in the ‘bioactive’ domain that binds with ErbB receptors, whereas our gene expression data shows that this region is intact in the *Nrg1* TM HET mouse, as discussed earlier. Other in vitro studies demonstrate that overexpression of type I Nrg1 reduces the frequency of carbachol-induced gamma oscillations [76], although it is difficult to compare changes in frequency from in vitro preparations to changes in power observed in our mouse model. Furthermore, overexpression of type I Nrg1 may lead to developmental changes over the postnatal lifespan that result in different effects to the acute exposure to Nrg1 that occurred in the Fisahn and Anderson studies, making it difficult to directly compare these two mouse models. In light of this literature, our data seem most consistent with an overexpression of Nrg1, such that baseline oscillations are increased, as previously observed with Nrg1 stimulus *in vitro*.

The relationship between Nrg1 and the molecular machinery underlying interneuron function and the generation of gamma oscillations requires further study. We observed no change in baseline levels of NMDA receptor mRNA; however, we found that the Nrg1 TMD mutation may influence the functional activation of NMDA receptors, by robustly decreasing cortical phosphorylation of Y1472 on the NR2B subunit in mutant mice compared to wild type-like mice, in agreement with a previous study [56]. The Y1472 residue is the major phosphorylation site of the NR2B subunit [77], and phosphorylation prevents internalisation of the NMDAR receptor, thereby prolonging its synaptic availability for activation and enhancing its cell surface function [78]. Disruptions to NMDAR phosphorylation may have a profound effect on NMDA receptor function and neuronal connectivity and lead to the generation of aberrant neural oscillations. However, the exact mechanism of the effect of the *Nrg1* TM mutation on NMDARs is still hard to pinpoint. One interpretation is that reduced NR2B phosphorylation is consistent with a situation of reduced Nrg1 function, since Nrg1-ErbB4 signalling activates the Fyn-Pyk2 pathway, which increases the phosphorylation of the NR2B subunit [56]. On the other hand, increased Nrg1-ErbB4 signalling over a prolonged developmental period may lead to a down-regulation of the availability or sensitivity of ErbB4 receptors and a reduced participation in this signalling, which could lead to reduced NR2B phosphorylation upon stimulation.

Further study of the molecular and behavioural phenotypes arising from Nrg1-ErbB4 manipulation, particularly in early stages of development, will shed light on the many questions that remain regarding Nrg1 and neuronal function. The relationship between altered gamma oscillations and the behavioural phenotype of *Nrg1* TM HET mice is still unclear, but the reduced gamma power may be pertinent to the working memory deficits previously identified in *Nrg1* TM HET mice [25], since gamma oscillations have been directly linked with working memory [79,80]. We observed a baseline hyperlocomotor phenotype in *Nrg1* TM HET mice, and an equivalent hyperlocomotor response to ketamine in *Nrg1* TM HET and wild type-like mice, replicating previous findings [10,31,33,81]. So far, research findings emphasise that Nrg1-ErbB4 mouse models are useful tools in schizophrenia research, including gain-of-function models for Nrg1 [82] but further investigation and integration of various approaches is required.

In conclusion, we present evidence that the *Nrg1* TM HET mouse is not a traditional gene ‘knock-out’ model of *Nrg1* haploinsufficiency, and we suggest that our findings are consistent with a gain of function model, aligned with the finding of increased NRG1 in the brains of human patients with schizophrenia [6]. We suggest exercising caution when interpreting data from *Nrg1* TM HET mice, bearing in mind that abnormalities might be a result of imbalanced Nrg1-ErbB signalling, rather than a simple increase or a decrease in Nrg1 function. The increased ongoing, and reduced auditory-evoked gamma frequency ECoG activity in the *Nrg1* TM HET mouse add to the existing evidence of schizophrenia-relevant molecular, behavioural and cognitive alterations in this mouse, and may represent a neural mechanism by which altered Nrg1 signalling may contribute to the pathogenesis of schizophrenia.

## Supporting Information

S1 Fig Geometric mean of Tbp and Ubc mRNA and 18S rRNA expression in hippocampus and prelimbic cortex of *Nrg1* TM HET mice and WT controls determined by qPCR (y-axis, mean (± S.E.M.) expression) plotted by postnatal day. n = 16–24.
(TIFF)

S2 Fig Western blot image of standard dilution series of 1–40 ug protein extracted from hippocampus of *Nrg1* TM HET mice and WT controls at postnatal day 35 using a C-terminal Nrg1 antibody (sc-348). A molecular weight ladder is shown on the left of this figure.
(TIF)

S1 Table Alignment of PCR products amplified from cDNA from hippocampus of female Nrg1 TM HET mice and WT controls at postnatal day 49 with NCBI reference sequence NM_178591.2. (DOCX)
