# Supplementary figures and images for: Neuregulin 1 Expression and Electrophysiological Abnormalities in the Neuregulin 1 Transmembrane Domain Heterozygous Mutant Mouse
Source: PLoS One. 2015 May 19;10(5):e0124114. doi: 10.1371/journal.pone.0124114 (PMC4437646; doi:10.1371/journal.pone.0124114)

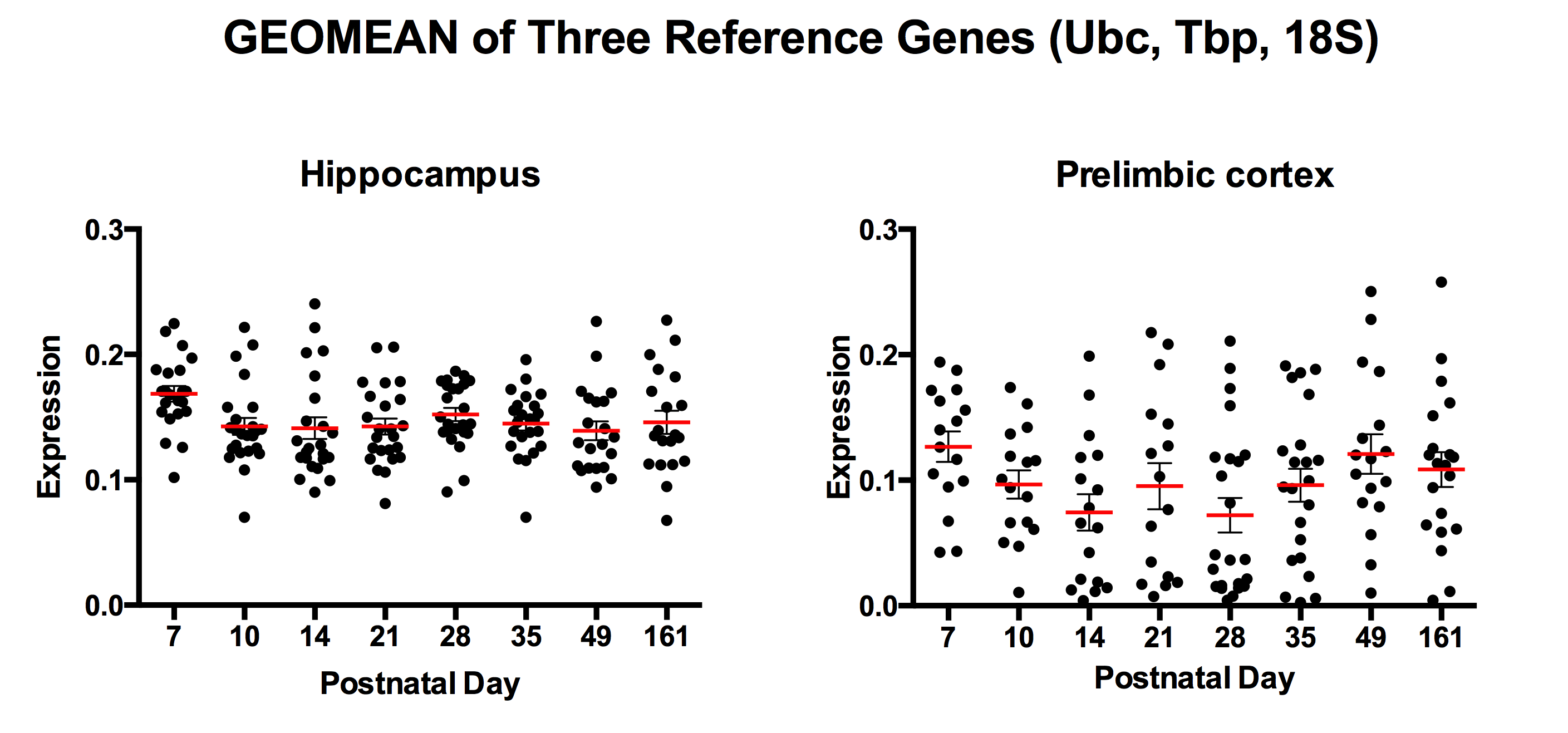

Supplement: S1 Fig — n = 16–24. (TIFF) [file pone.0124114.s001.tiff]

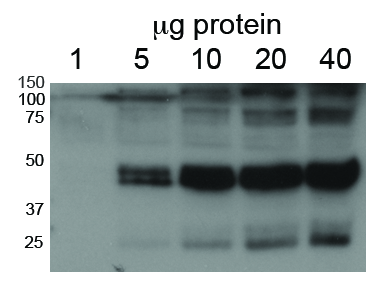

Supplement: S2 Fig — A molecular weight ladder is shown on the left of this figure. (TIF) [file pone.0124114.s002.tif]
