# Supplementary material for: Neuregulin 1 Expression and Electrophysiological Abnormalities in the Neuregulin 1 Transmembrane Domain Heterozygous Mutant Mouse
Source: PLoS One. 2015 May 19;10(5):e0124114. doi: 10.1371/journal.pone.0124114 (PMC4437646; doi:10.1371/journal.pone.0124114)
Supplement: S1 Table — (DOCX) [file pone.0124114.s003.docx]

S1 Table

| Sample | Genotype | Sequence |
| --- | --- | --- |
| 1 | WT | 10 20 30 40 50 60  SAMPLE GGAGCGGAGGAGCTCTACCAGA-GAGGGTACTGACAATTACTGGCATCTGTATCGCCCTG  : :::::::::::::::::::: :::::::::::::::::::::::::::::::::::::  NM_178591.2 GAAGCGGAGGAGCTCTACCAGAAGAGGGTACTGACAATTACTGGCATCTGTATCGCCCTG  880 890 900 910 920 930  70 80 90 100 110 120  SAMPLE TTGGTGGTCGGCATCATGTGTGTGGTGGCCTACTGCAAAACCAAGAAACAGCGGCAGAAG  ::::::::::::::::::::::::::::::::::::::::::::::::::::::::::::  NM_178591.2 TTGGTGGTCGGCATCATGTGTGTGGTGGCCTACTGCAAAACCAAGAAACAGCGGCAGAAG  940 950 960 970 980 990  130 140 150 160 170 180  SAMPLE CTTCATGATCGGCTCCGGCAGAGCCTTCGGTCAGAACGAAACAACATGGTGAACATAGCG  ::::::::::::::::::::::::::::::::::::::::::::::::::::::::::::  NM_178591.2 CTTCATGATCGGCTCCGGCAGAGCCTTCGGTCAGAACGAAACAACATGGTGAACATAGCG  1000 1010 1020 1030 1040 1050  190  SAMPLE AATGGCCCTCAC  ::::::::::::  NM_178591.2 AATGGCCCTCAC  1060 1070 |
| 2 | WT | 10 20 30 40 50 60  SAMPLE GGGAGCGGAG-AGCTCTACCAGA-GAGGGTACTGACAATTACTGGCATCTGTATCGCCCT  :: :::::::::::::::::::: ::::::::::::::::::::::::::::::::::::  NM_178591.2 GGAAGCGGAGGAGCTCTACCAGAAGAGGGTACTGACAATTACTGGCATCTGTATCGCCCT  880 890 900 910 920 930  70 80 90 100 110 120  SAMPLE GTTGGTGGTCGGCATCATGTGTGTGGTGGCCTACTGCAAAACCAAGAAACAGCGGCAGAA  ::::::::::::::::::::::::::::::::::::::::::::::::::::::::::::  NM_178591.2 GTTGGTGGTCGGCATCATGTGTGTGGTGGCCTACTGCAAAACCAAGAAACAGCGGCAGAA  940 950 960 970 980 990  130 140 150 160 170 180  SAMPLE GCTTCATGATCGGCTCCGGCAGAGCCTTCGGTCAGAACGAAACAACATGGTGAACATAGC  ::::::::::::::::::::::::::::::::::::::::::::::::::::::::::::  NM_178591.2 GCTTCATGATCGGCTCCGGCAGAGCCTTCGGTCAGAACGAAACAACATGGTGAACATAGC  1000 1010 1020 1030 1040 1050  190  SAMPLE GAATGGCCCTCAC  :::::::::::::  NM_178591.2 GAATGGCCCTCAC  1060 1070 |
| 3 | HET | 10 20 30 40 50 60  SAMPLE TTGGAGCGGAGGAGCTCTACCAGAAGAGGGTACTGACAATTACTGGCATCTGTATCGCCC  : : :::::::::::::::::::: :::::::::::::::::::::::::::::::::::  NM_178591.2 TGGAAGCGGAGGAGCTCTACCAGAAGAGGGTACTGACAATTACTGGCATCTGTATCGCCC  880 890 900 910 920 930  70 80 90 100 110 120  SAMPLE TGTTGGTGGTCGGCATCATGTGTGTGGTGGCCTACTGCAAAACCAAGAAACAGCGGCAGA  ::::::::::::::::::::::::::::::::::::::::::::::::::::::::::::  NM_178591.2 TGTTGGTGGTCGGCATCATGTGTGTGGTGGCCTACTGCAAAACCAAGAAACAGCGGCAGA  940 950 960 970 980 990  130 140 150 160 170 180  SAMPLE AGCTTCATGATCGGCTCCGGCAGAGCCTTCGGTCAGAACGAAACAACATGGTGAACATAG  ::::::::::::::::::::::::::::::::::::::::::::::::::::::::::::  NM_178591.2 AGCTTCATGATCGGCTCCGGCAGAGCCTTCGGTCAGAACGAAACAACATGGTGAACATAG  1000 1010 1020 1030 1040 1050  190  SAMPLE CGAATGGCCCTCAC  ::::::::::::::  NM_178591.2 CGAATGGCCCTCAC  1060 1070 |
| 4 | HET | 10 20 30 40 50 60  SAMPLE TACGGGAGGCGAGGAGCTCTACCAGAAGAGGGTACTGACAATTACTGGCATCTGTATCGC  :: :: ::::::::::::::::::::::::::::::::::::::::::::::::::::::  NM_178591.2 TATGGAAGCGGAGGAGCTCTACCAGAAGAGGGTACTGACAATTACTGGCATCTGTATCGC  880 890 900 910 920 930  70 80 90 100 110 120  SAMPLE CCTGTTGGTGGTCGGCATCATGTGTGTGGTGGCCTACTGCAAAACCAAGAAACAGCGGCA  ::::::::::::::::::::::::::::::::::::::::::::::::::::::::::::  NM_17859.12 CCTGTTGGTGGTCGGCATCATGTGTGTGGTGGCCTACTGCAAAACCAAGAAACAGCGGCA  940 950 960 970 980 990  130 140 150 160 170 180  SAMPLE GAAGCTTCATGATCGGCTCCGGCAGAGCCTTCGGTCAGAACGAAACAACATGGTGAACAT  ::::::::::::::::::::::::::::::::::::::::::::::::::::::::::::  NM_178591.2 GAAGCTTCATGATCGGCTCCGGCAGAGCCTTCGGTCAGAACGAAACAACATGGTGAACAT  1000 1010 1020 1030 1040 1050  190 200  SAMPLE AGCGAATGGCCCTCACCA  ::::::::::::::::::  NM_178591.2 AGCGAATGGCCCTCACCA  1060 1070 |
